# The Impact of Anticoagulation in Patients With Isolated Cancer‐Associated Splanchnic Vein Thrombosis: A Dual‐Center Cohort Study

**DOI:** 10.1002/ajh.70287

**Published:** 2026-03-27

**Authors:** Abhilasha Borad, Michael Andersen, Danielle Guffey, Hanqing Shang, Jun Y. Jiang, Maria J. Fernandez Turizo, Jonathan Berry, Jeffrey I. Zwicker, Rushad Patell, Ang Li

**Affiliations:** ^1^ Department of Medicine Beth Israel Deaconess Medical Center, Harvard Medical School Boston Massachusetts USA; ^2^ Division of Hematology‐Oncology University of Washington, Fred Hutchinson Cancer Center Seattle WA USA; ^3^ Institute for Clinical & Translational Research, Baylor College of Medicine Houston Texas USA; ^4^ Division of Hematology‐Oncology Keck School of Medicine of University of Southern California Los Angeles California USA; ^5^ Los Angeles General Medical Center Los Angeles California USA; ^6^ Hematology Service, Department of Medicine Memorial Sloan Kettering Cancer Center New York New York USA; ^7^ Division of Hematology and Hematologic Malignancies, Department of Medicine Beth Israel Deaconess Medical Center, Harvard Medical School Boston Massachusetts USA; ^8^ Weill Cornell Medical College New York New York USA; ^9^ Section of Hematology‐Oncology, Department of Medicine Baylor College of Medicine Houston Texas USA

**Keywords:** anticoagulants, hemorrhage, neoplasms, portal vein, venous thrombosis

## Abstract

Data to guide management of isolated bland cancer‐associated splanchnic vein thrombosis (CA‐SpVT) are limited. We aimed to assess the role of anticoagulation (AC) and bleeding and thrombosis in patients with CA‐SpVT. We conducted a dual‐center retrospective cohort study of adults with incident, isolated, bland CA‐SpVT from 2011 to 2020. The primary outcome was major bleeding (MB); other outcomes included usual‐site venous thromboembolism (VTE) recurrence and progression/recanalization of CA‐SpVT. Time‐to‐event outcomes were analyzed with weighted Cox models adjusting for cancer type, stage, SpVT location, and whether symptomatic. For SpVT recanalization/progression, differences were estimated using weighted average treatment effects (ATEs). After excluding tumor thrombus, we included 437 patients with notable characteristics of median age 60 years, portal vein thrombosis (81.2%), and underlying hepatocellular cancer (35.9%). Of these, 29.5% received therapeutic AC. At 6 months, there were 11.9% MB and 6.4% incident usual‐site VTE events. Among 308 patients with follow‐up imaging, the 1‐year thrombus progression rate was 19.8% and thrombus recanalization was 28.6%. In the adjusted analysis, there were numerically higher rates of MB with AC (adjusted hazard ratio [aHR] 1.93, 95% confidence interval [CI] 0.97–3.87) and no significant difference in the incidence of VTE (aHR 1.41, 95% CI 0.56–3.51). AC was associated with significantly higher likelihood of venous recanalization (ATE +24% 95% CI 13%–35%) and significantly lower likelihood of thrombus progression (ATE –14% 95% CI –23% to −5%). In patients with isolated bland CA‐SpVT, AC was associated with thrombus recanalization and limited thrombus progression; effects were offset by a potentially higher risk of MB.

## 1 Introduction

Venous thromboembolism (VTE) is a frequently diagnosed complication of malignancy, with an estimated 20%–30% of all initial VTE cases occurring in patients with cancer [1, 2, 3, 4]. Splanchnic vein thrombosis (SpVT), which includes hepatic, portal, mesenteric, and splenic vein thrombosis, is a less common manifestation of VTE that is often associated with cirrhosis, myeloproliferative disorders, and solid cancers, namely gastrointestinal and hepatobiliary malignancies [5, 6, 7, 8, 9, 10, 11]. Cancer‐associated SpVT (CA‐SpVT) carries an increased risk of thrombus extension, thrombus recurrence, and bleeding compared to noncancer‐associated thromboses [6, 11]. Additionally, the highest mortality rates from SpVT are seen in patients with solid malignancies when compared to other predisposing conditions [5, 12]. Moreover, treatment of CA‐SpVT is often challenging, as neoplasms are associated with their own set of unique complications including increased risk of bleeding, as well as increased risk of usual and atypical site thrombosis [6, 13, 14, 15, 16].

There is a paucity of high‐quality data in this space and existing data is based on single‐center observational and retrospective studies. Professional society guidelines reflect the uncertainty of optimal management of CA‐SpVT [11]. The International Society on Thrombosis and Hemostasis (ISTH) recommends treatment of symptomatic SpVT in patients regardless of cancer status based on weak quality of evidence, the American Society of Hematology recommends either observation or a short course of anticoagulation (AC) treatment in CA‐SpVT, and the American Society of Clinical Oncology recommends treatment of incidental CA‐SpVT on a case‐by‐case basis [17, 18, 19]. Thus, initiating AC in patients with CA‐SpVT can be a clinical challenge and requires nuanced considerations to balance the bleeding and thrombotic risks unique to this population.

Accurate estimates of bleeding risk and thrombotic outcomes associated with AC in this high‐risk population are needed to inform clinical decision making and future studies. We therefore conducted a dual‐center retrospective study to assess the association between AC and bleeding and thrombotic outcomes in patients with isolated, bland CA‐SpVT. Although each cohort has been previously reported independently, we intentionally combined these datasets to address a clinically important question that could not be adequately answered in single‐center analyses: the efficacy and potential harm of AC in patients with isolated, bland cancer‐associated SpVT without concurrent pulmonary embolism (PE) or deep vein thrombosis (DVT) [11, 20]. Pooling cohorts increased statistical power through a larger sample size, enabled robust confounder adjustment using propensity score (PS) weighting to account for treatment indication bias, and improved cohort diversity by incorporating inherent differences in patient race and ethnicity as well as institutional practice patterns in the management of SpVT. This harmonized dual‐center approach allowed a more rigorous and generalizable assessment of bleeding and thrombotic outcomes associated with AC in this high‐risk population.

## 2 Methods

### 2.1 Study Design

We conducted a dual‐center retrospective cohort study conducted across tertiary academic centers in Boston, Massachusetts (Beth Israel Deaconess Medical Center [BIDMC]) and Houston, Texas (Harris Health System [HHS]). Cohorts at these study sites have been previously individually published [11, 20]. Inclusion criteria were new SpVT diagnosed 2011–2022 in adult patients (age ≥ 18) with active solid cancer or lymphoma diagnosed or treated within the last 6 months and at least 3 months of follow‐up. SpVT diagnosis was made radiographically. We excluded patients if SpVT occurred > 6 months before or > 36 months after cancer diagnosis, myeloproliferative neoplasms (MPNs) and leukemias, concurrent usual site VTE including DVT or PE within 7 days of CA‐SpVT diagnosis, baseline use of AC prior to CA‐SpVT diagnosis, and radiographically diagnosed tumor thrombus or mixed tumor/bland thrombus. Cancer status was defined as active at the time of SpVT diagnosis if the diagnosis had been made within the past 6 months, if the cancer was metastatic, or if patient was receiving cancer‐directed treatment at the time of SpVT diagnosis.

Eligible patients with CA‐SpVT were identified at BIDMC using International Classification of Diseases (ICD) codes alone, whereas at HHS, case identification incorporated both ICD codes and a natural language processing (NLP) algorithm to extract relevant keywords from radiology reports [20, 21, 22]. Study investigators subsequently conducted manual chart review for all patients to confirm eligibility criteria were met. Additional information regarding patient characteristics, malignancy types, treatment strategies, and outcomes was also collected manually. No standardized institutional management protocol for CA‐SpVT was in place at participating centers, and treatment decisions were made at the discretion of the treating clinicians according to routine clinical practice. Patients were censored from the analysis at last clinical follow‐up.

### 2.2 Outcomes

The primary outcome was major bleeding (MB) as defined by the ISTH criteria [23]. Secondary outcomes included clinically‐relevant non‐major bleeding (CRNMB), clinically‐relevant bleeding (CRB, defined as either MB or CRNMB), usual‐site VTE, progression/recanalization of CA‐SpVT, and overall mortality. All outcomes except CA‐SpVT progression were assessed clinically for 6 months after CA‐SpVT diagnosis. Progression/recanalization was only assessed in a subset of patients with repeat radiologic abdominal scans. Specifically, CA‐SpVT progression was defined as either a new interval thrombus noncontiguous with the initial thrombus or an extension of thrombus contiguously into a new vein [11]. Recanalization was defined as a complete or partial resolution of the previously noted thrombus. Radiologic progression was defined based on imaging findings alone, and the presence or absence of associated clinical symptoms was not systematically collected or adjudicated.

### 2.3 Analysis

Baseline patient characteristics were compared between AC and non‐anticoagulation (non‐AC) groups. To estimate average treatment effect (ATE) associated with AC for each outcome, inverse probability of treatment weighting (IPTW) was used to account for measured confounders including cancer type, cancer stage, SpVT location, and whether index SpVT was symptomatic. Overlap weight was estimated by fitting PS logistic regression models on treatment assignment using baseline covariates via the *PSweight* package [24, 25]. The PS analysis was performed using all patient data because none of the included covariates had missingness. Covariate balance after PS weighting was checked via standardized mean difference (SMD) where an SMD of < 0.10 was considered adequate balance. For MB, CRNMB, usual‐site VTE, and mortality (time‐to‐event), we used unweighted Kaplan–Meier (KM) estimator (unadjusted) and weighted Cox regression model with robust standard errors (adjusted) [26, 27]. Absolute percentages, hazard ratios (HRs), and 95% confidence intervals (CIs) for each outcome at 180 days were reported. For CA‐SpVT progression/recanalization (binary outcome), we used unweighted and weighted ATE to assess the risk difference directly from the PS model. We chose the ATE framework for these binary outcomes to reflect an imaging‐defined disease state assessed during follow‐up rather than a discrete clinical event with a precisely observed time of onset. The timing of progression or recanalization is dependent on surveillance practices and thus interval censored, which limits the interpretability of time‐to‐event modeling.

In a sensitivity analysis, we repeated the above analysis after excluding patients with hepatocellular carcinoma (HCC) diagnosis due to severe imbalance in the rate of AC initiation for HCC versus non‐HCC cancer types. All statistical analyses were performed in R (Vienna, Austria 4.2.2).

## 3 Results

### 3.1 Patient Characteristics

A total of 437 patients (277 from BIDMC and 160 from HHS) with incident, isolated, bland CA‐SpVT were included in the final analytic cohort (Table 1). There were notable differences in patient characteristics between the two cohorts (Table S1). When combined, patients had a median age of 60 years (IQR 54–68), 57.7% were male, and 73.7% were non‐Hispanic. The racial breakdown included 71.9% White, 14.2% Black, 5.9% Asian Pacific Islander, and 8.0% other. The most common tumor types were HCC (*n* = 157, 35.9%), pancreatic (*n* = 90, 20.6%), colorectal (*n* = 47, 10.8%), and biliary (*n* = 43, 9.84%). Metastatic disease was present in 42.6% (*n* = 186) of patients. The majority (*n* = 266, 60.9%) of SpVT were asymptomatic. Most SpVT occurred in portal veins (*n* = 355, 81.2%), followed by mesenteric veins (*n* = 113, 25.9%), and splenic vein (*n* = 71, 16.3%).

**TABLE 1:** Baseline patient characteristics.

Covariate	Overall, *N* = 437	No anticoagulation, *N* = 308 (70.5%)	Anticoagulation, *N* = 129 (29.5%)	*p*
Site				
Beth Israel Deaconess Medical Center	277 (63.4%)	188 (61.0%)	89 (69.0%)	0.13
Harris Health System	160 (36.6%)	120 (39.0%)	40 (31.0%)	
Age at Diagnosis, years (range)	60 (54–68)	61 (54–67.5)	60 (52–69)	0.61
Male	252 (57.7%)	185 (60.1%)	67 (51.9%)	0.14
Race				
White	314 (71.9%)	213 (69.2%)	101 (78.3%)	0.22
Black	62 (14.2%)	47 (15.3%)	15 (11.6%)	
Asian/Asian Pacific Islander	26 (5.9%)	19 (6.2%)	7 (5.4%)	
Other	35 (8.0%)	29 (9.4%)	6 (4.7%)	
Ethnicity				
Non‐Hispanic	322 (73.7%)	219 (71.1%)	103 (79.8%)	0.074
Hispanic	115 (26.3%)	89 (28.9%)	26 (20.2%)	
Cancer type				
Hepatocellular	157 (35.9%)	139 (45.1%)	18 (14.0%)	< 0.001
Pancreatic	90 (20.6%)	57 (18.5%)	33 (25.6%)	
Biliary	43 (9.8%)	30 (9.7%)	13 (10.1%)	
Upper luminal GI	18 (4.1%)	12 (3.9%)	6 (4.7%)	
Lower luminal GI	47 (10.8%)	24 (7.8%)	23 (17.8%)	
Other solid	82 (18.8%)	46 (14.9%)	36 (27.9%)	
Metastatic disease	186 (42.6%)	121 (39.3%)	65 (50.4%)	0.034
Systemic cancer‐directed therapy	127 (29.1%)	82 (26.6%)	45 (34.9%)	0.085
Symptomatic	171 (39.1%)	107 (34.7%)	64 (49.6%)	0.005
Thrombus location				
Portal vein	355 (81.2%)	255 (82.8%)	100 (77.5%)	0.23
Mesenteric vein	113 (25.9%)	60 (19.5%)	53 (41.1%)	< 0.001
Splenic vein	71 (16.2%)	48 (15.6%)	23 (17.8%)	0.57

Abbreviations: DOAC: direct oral anticoagulation; GI: Gastrointestinal; LMWH: low molecular weight heparin; VKA: vitamin K antagonist.

At the time of CA‐SpVT diagnosis, 129 patients (29.5%) received therapeutic AC, with the most common agent being low molecular weight heparin (LMWH, *n* = 69, 53.5%) followed by direct oral anticoagulant (DOAC, *n* = 33, 25.6%). The median duration of AC was 90 days (IQR 60–181); the 68 LMWH users had a median duration of 90 days, while the 33 DOAC users and 27 VKA users both had a median duration of 120 days. Given the retrospective design, anticoagulant intensity and prescriber intent were not formally adjudicated. Long‐term therapeutic intent was inferred from initiation of therapeutic‐dose AC with continuation beyond hospitalization or at early outpatient follow‐up; brief courses of parenteral AC alone were not considered long‐term therapeutic treatment. Significant baseline differences were identified in cancer type, metastatic disease, symptomatic presentation, and thrombus location between the AC and non‐AC groups. HCC was present in 45.1% of patients in the non‐AC group versus 14.0% of patients in the AC group (*p* < 0.001) (Table S2). Metastatic disease was present in 50.4% of patients in the AC group versus 39.3% of patients in the non‐AC group (*p* = 0.034). Presentation was symptomatic in 49.6% of patients in the AC group versus 34.7% of patients in the non‐AC group (*p* = 0.005). Thrombus was present in the mesenteric vein in 41.1% of patients in the AC group versus 19.5% of patients in the non‐AC group (*p* < 0.001). After PS weighting, the two groups were balanced with SMD of 0 for all covariates (Table S3).

### 3.2 Bleeding, Thrombotic, Progression/Recanalization, and Mortality Outcomes

**FIGURE 1:**
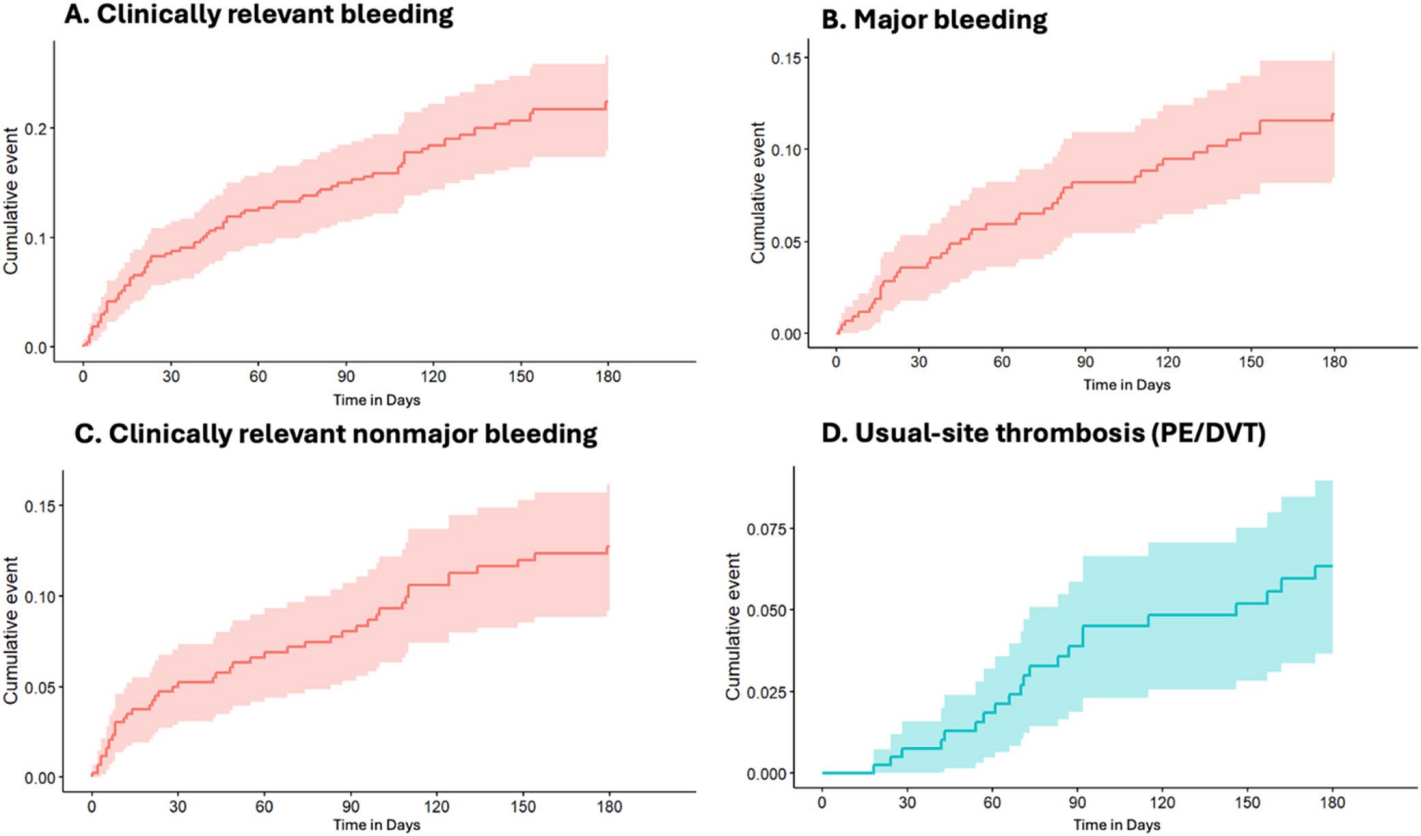
Cumulative incidence of clinical outcomes among patients with cancer‐associated splanchnic vein thrombosis (CA‐SpVT). Cumulative incidence curves showing (A) clinically relevant bleeding (CRB), (B) major bleeding (MB), (C) clinically relevant nonmajor bleeding (CRNMB), and (D) usual‐site venous thromboembolism following diagnosis of CA‐SpVT. [Color figure can be viewed at wileyonlinelibrary.com]

The primary and secondary outcomes are shown in Table 2. The overall bleeding and thrombotic outcomes were high in this cohort of patients with CA‐SpVT. By 6 months, MB occurred in 11.9% of patients (*n* = 43). CRNMB occurred in 12.7% (*n* = 46). Overall, CRB occurred in 22.4% (*n* = 83), with categories not mutually exclusive (Figure 1A–C). Among patients receiving AC, MB events occurred throughout follow‐up without clear early clustering (five events within 30 days, four between days 31 and 60, four between days 61 and 90, and five between days 91 and 180). In contrast, CRNMB events occurred predominantly early after AC initiation, with 10 of 13 (77%) occurring within the first 30 days. The main site of MB was gastrointestinal (64.7%), followed by brain (11.8%). Similarly, the main site of CRNMB was gastrointestinal (76.5%). In contrast, there were 6.4% incident usual‐site VTE events (*n* = 21) and 37.3% mortality (*n* = 161) (Figure 1D). Of the 10 patients receiving AC who developed usual‐site VTE, five developed while on AC and five developed after AC was discontinued. Additionally, nine patients experienced MB while on AC and this led to permanent cessation of AC, while several patients (*n* = 4) had concurrent bleeding and VTE complications regardless of their AC exposure. In the subset of patients with follow‐up abdominal scans (*n* = 308), the 1‐year incidence of thrombus recanalization was 28.6% (*n* = 88) and that of thrombus progression was 19.8% (*n* = 61). When stratified by study site, site‐specific 6‐month cumulative incidences of bleeding, thrombotic events, and mortality, as well as 12‐month rates of venous recanalization and progression, were estimated (Table S4).

**TABLE 2:** Primary and secondary outcomes before and after propensity score weighting.

All patients	Number of patients	Number of events	Before PS weight			After PS weight		
			HR (95% CI)	−AC	+AC	aHR (95% CI)	−AC	+AC
Clinically‐relevant bleeding (CRB)^a^	437	83	1.28 (0.82, 2.01)	20.9%	25.6%	1.31 (0.80, 2.16)	21.2%	26.9%
Major bleeding (MB)^a^	437	43	1.69 (0.92, 3.09)	9.8%	16.8%	1.93 (0.97, 3.87)	9.3%	17.7%
Clinically‐relevant non‐major bleeding (CRNMB)^a^	437	46	0.92 (0.48, 1.75)	13.4%	11.1%	0.79 (0.39, 1.59)	14.7%	11.8%
Usual‐site VTE^a^	437	21	2.11 (0.90, 4.98)	4.8%	9.9%	1.41 (0.56, 3.51)	6.6%	9.1%
Mortality^a^	437	161	0.91 (0.65, 1.29)	37.6%	36.5%	0.80 (0.56, 1.17)	41.6%	35.2%

Subset with scans	Number of patients	Number of events	ATE (95% CI)	−AC	+AC	ATE (95% CI)	−AC	+AC
Thrombus recanalization^b^	308	88	+19% (0.08, 0.30)	22.6%	41.7%	+24% (0.13, 0.35)	20.1%	44.3%
Thrombus progression^b^	308	61	−12% (−0.22, −0.03)	23.6%	11.5%	−14% (−0.23, −0.05)	21.4%	10.3%

Abbreviations: AC: anticoagulation; aHR: adjusted hazard ratio; ATE: average treatment effect; CI: confidence interval; HR: hazard ratio; PE/DVT: pulmonary embolism or deep vein thrombosis; PS: propensity score.

^a^ Over 6‐month period.

^b^ Over 12‐month period.

After performing PS weight to balance baseline characteristics in the overall cohort of 437 patients, AC was associated with an adjusted HR of 1.31 (95% CI 0.80–2.16) for CRB (26.9% +AC vs. 21.2% −AC) and HR of 1.93 (95% CI 0.97–3.87) for MB (17.7% +AC vs. 9.3% −AC). In contrast, AC was associated with an adjusted HR of 1.41 (95% CI 0.56–3.51) for usual‐site VTE (9.1% +AC vs. 6.6% −AC) and HR of 0.80 (95% CI 0.56–1.17) for mortality (35.2% vs. 41.6%). In the subset of patients with follow‐up abdominal scans, AC was associated with significantly increased probability of venous recanalization (ATE of +24% [95% CI 13%–35%]) and significantly decreased probability of thrombus progression (ATE of −14% [95% CI −23% to −5%]).

### 3.3 Sensitivity Analysis After Excluding Patients With HCC

Since most (88.5%) patients with HCC did not receive AC in our combined cohort, we performed a sensitivity analysis after excluding those patients. In the remaining 280 patients, we repeated PS weighting and found that AC was similarly associated with a numerically higher risk of CRB (HR of 1.21 [95% CI 0.68–2.16]) and MB (HR of 1.90 [95% CI 0.83–4.32]) (Table S5). Furthermore, AC was similarly associated with an increased chance of venous recanalization (ATE of +23% [95% CI 9%–38%]) and a decreased risk of thrombus progression (ATE of −14% [95% CI −26% to −3%]).

## 4 Discussion

AC has been shown to improve thrombotic outcomes, such as recanalization rates in patients with abdominal vein thrombosis, and prevent extension of the underlying thrombosis [28]. However, these data are derived from studies that included only a minority of patients with malignancy, although cancer is seen in up to one‐fifth of patients with unselected SpVT [29]. Furthermore, cancer is an extremely prothrombotic state associated with high rates of thrombotic recurrence after usual‐site VTE compared to patients without cancer [30, 31, 32, 33]. In this dual‐center study, we found that overall rates of thrombosis were substantial including high rates of abdominal vein thrombus progression (19.8%) as well as new usual‐site VTE (6%) at 6 months from the index abdominal vein thrombotic event. Although only about one‐third of the overall cohort received therapeutic AC for the CA‐SpVT, AC use was associated with significantly increased venous recanalization and significantly reduced the risk of thrombus progression in the splanchnic vasculature. We excluded patients with other indications of AC prior to the index CA‐SpVT and matched the cohort for known important risk factors of thrombosis in cancer (including cancer site and stage) [34], however, the observed numerically higher rates of usual‐site VTE (including lower extremity DVT and PE) with AC likely reflect residual confounding, as patients thought to be at higher baseline thrombotic risk were more likely to be prescribed AC. If this is the case, the observed increases in thrombus recanalization and reduction in progression may be an underestimate. Moreover, unmeasured variables such as personal or family history of VTE or cancer‐specific prothrombotic factors such as agents used, which were unaccounted for, may also have contributed to this observed difference. Other potential factors not explored in this study including insufficient treatment duration, use of subtherapeutic doses, and drug–drug interactions may have contributed to the observed high rates of usual‐site VTE. Additionally, the wide confidence intervals around the usual‐site VTE estimates limit interpretability of these findings.

MB in patients with cancer that received AC for 6 months for acute VTE in contemporary clinical trials has been reported to range from 4% to 7% [35, 36, 37]. Similarly, in a real‐world prospective cohort study of 791 ambulatory patients initiating systemic antineoplastic therapy, the 6‐month MB incidence was 5.1% (95% CI, 3.4–6.7) [38]. In our retrospective cohort study, in which over two‐thirds were not anticoagulated, we report a 6‐month rate of MB of 11.9%. These substantially high MB rates in patients with CA‐SpVT could be related to even higher risks stemming from the underlying malignancy (a population enriched for luminal gastrointestinal cancer and metastatic disease), complications of antineoplastic therapies (such as thrombocytopenia), portal hypertension leading to variceal bleeding, and systemic comorbidities and organ dysfunction (particularly cirrhosis in patients with HCC). The use of AC in this high‐risk cohort was associated with a doubling of MB rates, which needs to be carefully balanced with increased rates of revascularization.

In line with registry studies for SpVT, we found HCC as the most frequent underlying malignancy in our study [12, 39]. Chronic liver disease is seen in over 90% of patients with HCC [40], and sequelae include portal hypertension, hypersplenism, and impaired metabolism of anticoagulants leading to narrow therapeutic windows for frequently used anticoagulants such as DOACs and vitamin K antagonists [41]. These clinical considerations are likely reflected in the low rates of AC (15%) we observed in patients with HCC and SpVT. Given these inherent bleeding risks in patients with underlying liver disease, our subgroup analysis excluded patients with HCC but showed similar results to the overall cohort with regards to effects of AC on bleeding and venous recanalization and thrombus progression, suggesting that these effects were not driven by the underlying cancer type alone.

In patients with newly diagnosed CA‐SpVT, there is considerable equipoise with regards to AC with significant variations not just by patient factors (such as thrombotic burden, comorbidities, and underlying bleeding risk) but also by centers and provider preference. We combined data from two distinct US healthcare systems: HHS, a county hospital network serving predominantly uninsured, Spanish‐speaking patients with advanced and often screening‐preventable malignancies; and BIDMC, a private, academic tertiary‐care center serving largely insured, English‐speaking patients. The results of this integrated analysis were consistent with those observed in the individual cohorts while enabling adequately powered PS‐matched comparisons and yielded more precise effect estimates. Importantly, the consistency of findings across these structurally and demographically different populations strengthens the generalizability of the results and supports their applicability beyond a single health system context. However, integrating data from these diverse populations also introduced inherent heterogeneity that must be acknowledged when interpreting outcomes. Although data abstraction and verification were performed manually and uniformly across both sites, the markedly higher bleeding rates observed at HHS may reflect both a population with more advanced disease and greater comorbidities as well as potential methodological differences in the ascertainment and coding of bleeding events. Bleeding events are inherently more subjective than imaging‐confirmed thrombotic outcomes; although standardized definitions were utilized across sites, we did not train abstractors in parallel to ensure intercoder reliability [23, 42]. Adjustment for broader measures of disease severity and socioeconomic status or access to healthcare would likely limit this apparent difference, but such variables were not available in the current dataset.

Although we were able to create a sizeable combined dataset with harmonized inclusion and exclusion criteria and manually abstracted clinical thrombotic and bleeding outcomes to evaluate the role of AC in CA‐SpVT, there are limitations that could be addressed in future iterations. Importantly, AC agent, dosage, and duration are important modulators of bleeding and thrombotic risk, but our relatively small number of patients that received did not permit assessment of these factors or anticoagulant‐specific effects. Laboratory factors including thrombocytopenia are known to influence the risk of bleeding considerably; however, these data were not available uniformly for both sites, and thus we were unable to report laboratory AC parameters or account for these in our analysis. In addition, detailed characterization of gastrointestinal bleeding events as variceal versus nonvariceal was not systematically captured, which may be particularly relevant in patients with portal hypertension or HCC. We deliberately excluded patients with CA‐SpVT in whom the thrombus was believed to be partially or entirely composed of tumor rather than bland clot, as these lesions are thought to represent a distinct biologic process with different thrombotic potential and a less clearly defined role for AC in their management [43, 44]. Similarly, hematologic malignancies, particularly MPNs, are well‐established risk factors for thrombosis in atypical locations, especially within the abdominal venous system, but represent a distinct population with unique pathophysiologic and therapeutic considerations [45, 46]. Although patients with lymphoma were included in this analysis, the cohort predominantly comprised individuals with solid gastrointestinal malignancies. Accordingly, the findings should not be extrapolated to patients with hematologic malignancies, particularly MPN‐SpVT as these patients were excluded. Finally, outcomes were not adjudicated independently or blindly and we relied on data and findings in the clinical charts that were entered for routine clinical care. Thus, although we adhered to published guidance to identify and grade the thrombotic and bleeding outcomes, inaccuracies may still arise due to inherent limitations in retrospective chart review. Prospective multicenter studies could enable more uniform and granular data collection.

## 5 Conclusion

In this multicenter retrospective cohort study, long‐term AC treatment (average of 3 months) of isolated, bland CA‐SpVT was associated with significantly improved venous recanalization (+24%) and decreased thrombus progression (−14%), albeit at a cost of doubling risk of MB (9%–18%). Future studies are required to assess individualized risk factors for bleeding in this patient population. These data support a tailored approach to AC decision‐making in patients with isolated CA‐SpVT.

## Author Contributions

Conception and design: R.P. and A.L. Collection and assembly of data: H.S., J.Y.J., M.A., M.J.F., A.B., R.P. and A.L. Data analysis: D.G. and A.L. Data interpretation: M.A., A.B., R.P. and A.L. Manuscript writing: A.B., R.P. and A.L. Final approval of manuscript: all authors.

## Conflicts of Interest

R.P. reports consultancy for Merck Research and Sanofi. J.I.Z. received data safety monitoring board fees from Sanofi and CSL Behring; consultancy fees from Perceptive, Incyte, Bristol Myers Squibb (BMS), Regeneron; all other coauthors report no financial disclosures or potential sources of conflicts.

## Supporting information


**Table S1:** Patient characteristics by site.
**Table S2:** Patient characteristics by hepatocellular carcinoma (HCC).
**Table S3:** Patient characteristics before and after propensity score weighting (standardized mean difference).
**Table S4:** Study outcomes stratified by study site.
**Table S5:** Sensitivity analysis after exclusion of patients with hepatocellular carcinoma.

## Data Availability

The data that support the findings of this study are available from the corresponding author upon reasonable request.
