# Supplementary material for: The Impact of Anticoagulation in Patients With Isolated Cancer‐Associated Splanchnic Vein Thrombosis: A Dual‐Center Cohort Study
Source: Am J Hematol. 2026 Mar 27;101(6):1379–87. doi: 10.1002/ajh.70287 (PMC13107186; doi:10.1002/ajh.70287)
Supplement: Supplementary file 1 — Table S1: Patient characteristics by site. Table S2: Patient characteristics by hepatocellular carcinoma (HCC). Table S3: Patient characteristics before and after propensity score weighting (standardized mean difference). Table S4: Study outcomes stratified by study site. Table S5: Sensitivity analysis after exclusion of patients with hepatocellular carcinoma. [file AJH-101-1379-s001.docx]

**Supplemental Table 1: Patient Characteristics by Site**

| **Covariate** | **BCM/HHS**  **N=160 (36.6%)** | **BIDMC**  **N=277 (63.4%)** | **P-value** |
| --- | --- | --- | --- |
| **Age at Diagnosis, years (range)** | 56 (50-62) | 63 (56-70) | <0.001 |
| **Men** | 88 (55.0%) | 164 (59.2%) | 0.42 |
| **Race** |  |  |  |
| White | 120 (75.0%) | 194 (70.0%) | <0.001 |
| Black | 31 (19.4%) | 31 (11.2%) |  |
| Asian/Asian Pacific Islander | 8 (5.0%) | 18 (6.5%) |  |
| Other | 1 (0.6%) | 34 (12.3%) |  |
| **Ethnicity** |  |  |  |
| Non-Hispanic | 60 (37.5%) | 262 (94.6%) | <0.001 |
| Hispanic | 100 (62.5%) | 15 (5.4%) |  |
| **Cancer Type** |  |  |  |
| Hepatocellular | 46 (28.7%) | 111 (40.1%) | 0.016 |
| Pancreatic | 27 (16.9%) | 63 (22.7%) |  |
| Biliary | 17 (10.6%) | 26 (9.4%) |  |
| Upper Luminal GI | 7 (4.4%) | 11 (4.0%) |  |
| Lower Luminal GI | 24 (15.0%) | 23 (8.3%) |  |
| Other Solid | 39 (24.4%) | 43 (15.5%) |  |
| **Metastatic Disease** | 78 (48.8%) | 108 (39.0%) | 0.056 |
| **Systemic Cancer-Directed Therapy** | 61 (38.1%) | 66 (23.8%) | 0.002 |
| **Symptomatic** | 44 (27.5%) | 127 (45.8%) | <0.001 |
| **Thrombus Location** |  |  |  |
| Portal Vein | 106 (66.2%) | 249 (89.9%) | <0.001 |
| Mesenteric Vein | 49 (30.6%) | 64 (23.1%) | 0.090 |
| Splenic Vein | 38 (23.8%) | 33 (11.9%) | 0.002 |
| **Anticoagulation** | 40 (25%) | 89 (32.1%) | 0.13 |
| **Anticoagulation Duration, days** | 82 (33-223) | 90 (60-180) | 0.81 |
| **Anticoagulation Type** |  |  |  |
| None | 120 (75.0%) | 188 (67.9%) | 0.32 |
| LMWH | 24 (15.0%) | 45 (16.2%) |  |
| DOAC | 8 (5.0%) | 25 (9.0%) |  |
| VKA | 8 (5.0%) | 19 (6.9%) |  |

BCM/HHS: Baylor College of Medicine/Harris Health System; BIDMC: Beth Israel Deaconess Medical Center; GI: gastrointestinal; LMWH: low molecular weight heparin; DOAC: direct oral anticoagulant; VKA: Vitamin K antagonist

**Supplemental Table 2: Patient Characteristics by hepatocellular carcinoma (HCC)**

| **Covariate** | **No HCC**  **N=280 (64.1%)** | **HCC**  **N=157 (35.9%)** | **P-value** |
| --- | --- | --- | --- |
| **Site** |  |  | 0.018 |
| Beth Israel Deaconess Medical Center | 166 (59.3%) | 111 (70.7%) |  |
| Harris Health System | 114 (40.7%) | 46 (29.3%) |  |
| **Age at Diagnosis, years** | 59 (52-67) | 62 (56-68) | 0.005 |
| **Men** | 135 (48.2%) | 117 (74.5%) | <0.001 |
| **Race** |  |  |  |
| White | 214 (72.9%) | 100 (63.7%) | <0.001 |
| Black | 41 (14.6%) | 21 (13.4%) |  |
| Asian/Asian Pacific Islander | 12 (4.3%) | 14 (8.9%) |  |
| Other | 13 (4.6%) | 22 (14.0%) |  |
| **Ethnicity** |  |  |  |
| Non-Hispanic | 204 (72.9%) | 118 (75.2%) | 0.65 |
| Hispanic | 76 (27.1%) | 39 (24.8%) |  |
| **Metastatic Disease** | 162 (57.9%) | 24 (15.3%) | <0.001 |
| **Systemic Cancer-Directed Therapy** | 108 (38.6%) | 19 (12.1%) | <0.001 |
| **Symptomatic** | 119 (42.5%) | 52 (33.1%) | 0.066 |
| **Thrombus Location** |  |  |  |
| Portal Vein | 211 (75.4%) | 144 (91.7%) | <0.001 |
| Mesenteric Vein | 82 (29.3%) | 31 (19.7%) | 0.031 |
| Splenic Vein | 62 (22.1%) | 9 (5.7%) | <0.001 |
| **Anticoagulation** | 111 (39.6%) | 18 (11.5%) | <0.001 |
| **Anticoagulation Duration, days** | 90 (60-181) | 112 (60-180) | 0.90 |
| **Anticoagulation Type** |  |  |  |
| None | 169 (60.4%) | 139 (88.5%) | <0.001 |
| LMWH | 64 (22.9%) | 5 (3.2%) |  |
| DOAC | 33 (11.8%) | 0 (0.0%) |  |
| VKA | 14 (5.0%) | 13 (8.3%) |  |

HCC: hepatocellular carcinoma; LMWH: low molecular weight heparin; DOAC: direct oral anticoagulant; VKA: Vitamin K antagonist

**Supplemental Table 3: Patient characteristics before and after propensity score weighting (standardized mean difference)**

|  | **Unweighted** | | | **Weighted** | | |
| --- | --- | --- | --- | --- | --- | --- |
| **Characteristic** | **Mean No Anticoagulation** | **Mean Anticoagulation** | **SMD** | **Mean No Anticoagulation** | **Mean Anticoagulation** | **SMD** |
| Age, years | 60.536 | 59.837 | 0.061 | 59.790 | 59.790 | 0 |
| Male | 0.601 | 0.519 | 0.164 | 0.556 | 0.556 | 0 |
| Race White | 0.692 | 0.783 | 0.208 | 0.756 | 0.756 | 0 |
| Race Black | 0.153 | 0.116 | 0.106 | 0.124 | 0.124 | 0 |
| Race Asian | 0.062 | 0.054 | 0.032 | 0.060 | 0.060 | 0 |
| Hispanic | 0.289 | 0.202 | 0.204 | 0.230 | 0.230 | 0 |
| Tumor Hepatic | 0.451 | 0.140 | 0.725 | 0.205 | 0.205 | 0 |
| Tumor Pancreatic | 0.185 | 0.256 | 0.171 | 0.223 | 0.223 | 0 |
| Tumor biliary | 0.097 | 0.101 | 0.011 | 0.125 | 0.125 | 0 |
| Tumor Gastrointestinal | 0.117 | 0.225 | 0.289 | 0.196 | 0.196 | 0 |
| Tumor Metastatic | 0.393 | 0.504 | 0.224 | 0.495 | 0.495 | 0 |
| SpVT Symptoms | 0.347 | 0.496 | 0.304 | 0.438 | 0.438 | 0 |
| SpVT Portal | 0.828 | 0.775 | 0.132 | 0.793 | 0.793 | 0 |
| SpVT Mesenteric | 0.195 | 0.411 | 0.482 | 0.322 | 0.322 | 0 |
| SpVT Splenic | 0.156 | 0.178 | 0.060 | 0.166 | 0.166 | 0 |

SMD, standardized mean difference; SpVT, Splanchnic vein thrombosis

**Supplemental Table 4: Study outcomes stratified by study site**

| **All Patients** | **BCM/HHS**  **N=160 (36.6%)** | **BIDMC**  **N=277 (63.4%)** |
| --- | --- | --- |
| Major Bleeding (MB)* | 20 (12.5%) | 23 (8.3%) |
| Clinically- Relevant Non-Major Bleeding (CRNMB)* | 27 (16.88%) | 19 (6.9%) |
| Usual-Site PE/DVT* | 8 (5%) | 13 (4.69%) |
| Mortality | 60 (37.5%) | 101 (36.5%) |
| **Subset with scans** | **N=79** | **N=229** |
| Thrombus Recanalization^ | 20 (25.3%) | 68 (29.7%) |
| Thrombus Progression^ | 11 (13.9%) | 50 (21.8%) |

BCM/HHS: Baylor College of Medicine/Harris Health System; BIDMC: Beth Israel Deaconess Medical Center; MB: major bleeding; CRNMB: clinically-relevant non-major bleeding; PE/DVT: pulmonary embolism/deep vein thrombosis

*Over 6-month period

^Over 12-month period

**Supplemental Table 5: Sensitivity analysis after exclusion of patients with hepatocellular carcinoma**

|  |  |  | **Before PS Weight** | | | **After PS Weight** | | |
| --- | --- | --- | --- | --- | --- | --- | --- | --- |
| **All Patients** | **Number**  **Patients** | **Number**  **Events** | **HR**  **(95% CI)** | **-AC** | **+AC** | **aHR**  **(95% CI)** | **-AC** | **+AC** |
| Clinically-Relevant Bleeding (CRB)* | 280 | 51 | 1.32  (0.76, 2.29) | 20.5% | 24.2% | 1.21  (0.68, 2.16) | 22.0% | 26.0% |
| Major Bleeding (MB)* | 280 | 27 | 1.82  (0.85, 3.90) | 9.3% | 16.1% | 1.90  (0.83, 4.32) | 9.0% | 16.4% |
| Clinically-Relevant Non-Major Bleeding (CRNMB)* | 437 | 46 | 0.92  (0.48, 1.75) | 13.4% | 11.1% | 0.79  (0.39, 1.59) | 14.7% | 11.8% |
| Usual-Site PE/DVT* | 437 | 21 | 2.11  (0.90, 4.98) | 4.8% | 9.9% | 1.41  (0.56, 3.51) | 6.6% | 9.1% |
| Mortality* | 437 | 161 | 0.91  (0.65, 1.29) | 37.6% | 36.5% | 0.80  (0.56, 1.17) | 41.6% | 35.2% |
| **Subset with scans** | **Number**  **Patients** | **Number**  **Events** | **ATE**  **(95% CI)** | **- AC** | **+AC** | **ATE**  **(95% CI)** | **- AC** | **+AC** |
| Thrombus Recanalization^ | 308 | 88 | +19%  (0.08, 0.30) | 22.6% | 41.7% | +24%  (0.13, 0.35) | 20.1% | 44.3% |
| Thrombus Progression^ | 308 | 61 | -12%  (-.22, -0.03) | 23.6% | 11.5% | -14%  (-0.23, -0.05) | 21.4% | 10.3% |

PS: propensity score; HR: hazard ratio; aHR: adjusted hazard ratio; PE/DVT: pulmonary embolism/deep vein thrombosis; ATE: average treatment effect; AC: anticoagulation

*Over 6-month period

^Over 12-month period
